# Isolated Lesion of Penile Kaposi Sarcoma in an HIV-Negative Male Patient

**DOI:** 10.7759/cureus.88451

**Published:** 2025-07-21

**Authors:** Ananya Eeraveni, Gregory Gates, Arun Singh, Iris Ahronowitz

**Affiliations:** 1 Dermatology, University of Chicago Pritzker School of Medicine, Chicago, USA; 2 Dermatopathology, University of California Los Angeles David Geffen School of Medicine, Los Angeles, USA; 3 Oncology, University of California Los Angeles David Geffen School of Medicine, Los Angeles, USA; 4 Dermatology, University of California Los Angeles David Geffen School of Medicine, Los Angeles, USA

**Keywords:** genital kaposi sarcoma, hiv negative kaposi sarcoma, kaposi sarcoma, penile lesion, penile tumor

## Abstract

Kaposi sarcoma (KS) is a vascular neoplasm commonly associated with HIV positivity and immunocompromise. A subtype of KS has been more recently described in HIV-negative men who have sex with men (MSM). We report a case of a patient presenting with a solitary lesion on the penis, which was diagnosed to be KS. The patient was a 43-year-old male with a previous history of treatment with valacyclovir for herpes simplex virus 1 (HSV-1) who tested negative for multiple sexually transmitted infections, including HIV. Diagnosis of KS was confirmed through histopathology of a skin punch biopsy. While KS is mostly associated with HIV-positive and immunosuppressed patients, it is important to note that anyone who has been infected with human herpesvirus 8 (HHV-8), usually transmitted through bodily fluids, can develop KS. MSM may statistically be at higher risk for developing KS, even without HIV-positive status. Prompt diagnosis is important in guiding clinical management in these patients.

## Introduction

Kaposi Sarcoma (KS) is a low-grade vascular tumor with aggressive potential, historically divided into four epidemiological subtypes: HIV-associated, immunosuppression-associated, classic (typically seen in HIV-negative immunocompetent men of Mediterranean or Ashkenazi Jewish descent), and African-endemic. More recently, a fifth clinical subtype has been described, in HIV-negative immune immunocompetent men who have sex with men (MSM). The affected patients are typically younger and do not have the geographic or ethnic association described in classic KS [1]. In a case series of KS patients from a large cancer center, 11% of cases fit into this fifth subtype, of whom 55% had a solitary lesion at diagnosis and only 5.5% had genital involvement [2]. While KS can vary in clinical presentation, it is often described as solitary or multiple raised, reddish-purple papule(s) or nodule(s) on the skin or mucosa. Penile lesions are noted to be less common manifestations of KS, representing the initial manifestation of disease in less than 3% of cases [3]. 

Infection with human herpesvirus 8 [HHV-8, also known as Kaposi sarcoma-associated herpesvirus (KHSV)], which is transmitted through bodily fluid (predominantly saliva) exposure, is required for the development of KS. HHV-8 is common among MSM, with an overall estimated 33% seroprevalence in this population [4]. In the United States, KS prevalence is low and most commonly seen in HIV-positive individuals. We report a case of an HIV-negative MSM who presented with a solitary nonspecific penile lesion subsequently diagnosed as KS, highlighting the broad spectrum of KS presentation and the importance of considering the diagnosis of KS even in atypical and nonspecific presentations.

## Case presentation

**Figure 1:**
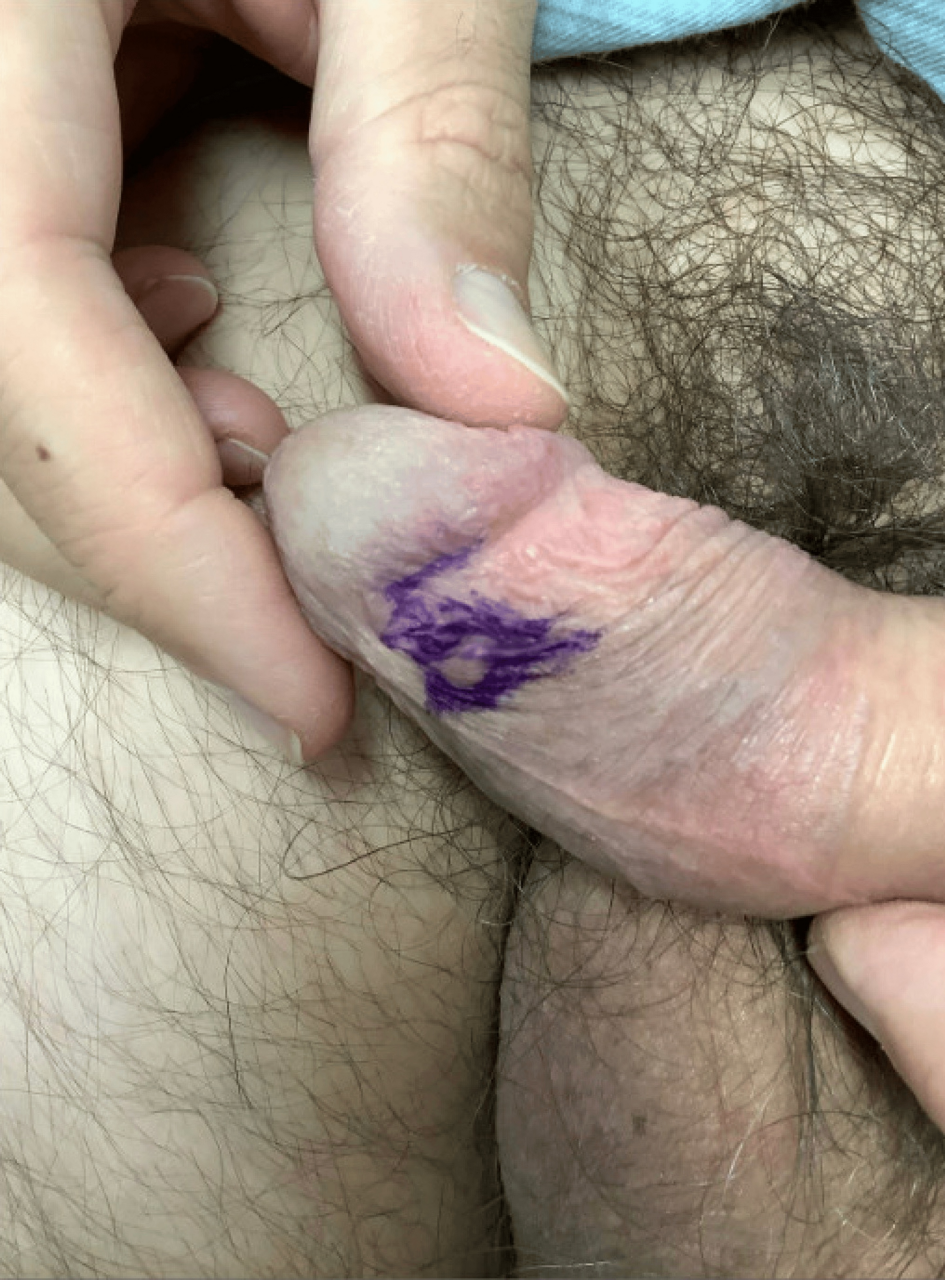
Skin colored papule on the ventral inner prepuce (found to be Kaposi sarcoma on histopathology)

A 43-year-old Argentinian male originally presented to the dermatology clinic for evaluation of an asymptomatic penile lesion that had been noted by the patient one month prior, with no ongoing growth observed. Upon physical examination, a 4 mm subcutaneous skin colored nodule resembling an epidermoid cyst was appreciated on the ventral inner prepuce, proximal to the glans (Figure 1). No ulceration was present. Dermoscopy revealed a featureless, skin-colored papule without a rainbow sign. No inguinal lymphadenopathy was noted.

His past medical history included orolabial herpes simplex virus 1 (HSV-1) on the left lower vermillion border of the lip with a history of frequent recurrence. The patient had been taking suppressive dosing of 500 mg of valacyclovir daily for several years but had stopped one to two months before the onset of the lesion. He had a normal complete blood count and tested negative for sexually transmitted infections, including syphilis, hepatitis B, hepatitis C, and HIV. He reported a recent episode of bleeding gums that occurred while brushing his teeth, which had since resolved. Physical examination did not indicate any gingival abnormality. He denied hemoptysis, hematochezia, or edema. He reported no personal history of skin cancer. The patient’s family history was positive for melanoma in his father. He reported a monogamous relationship with his male partner for the last six years.

**Figure 2:**
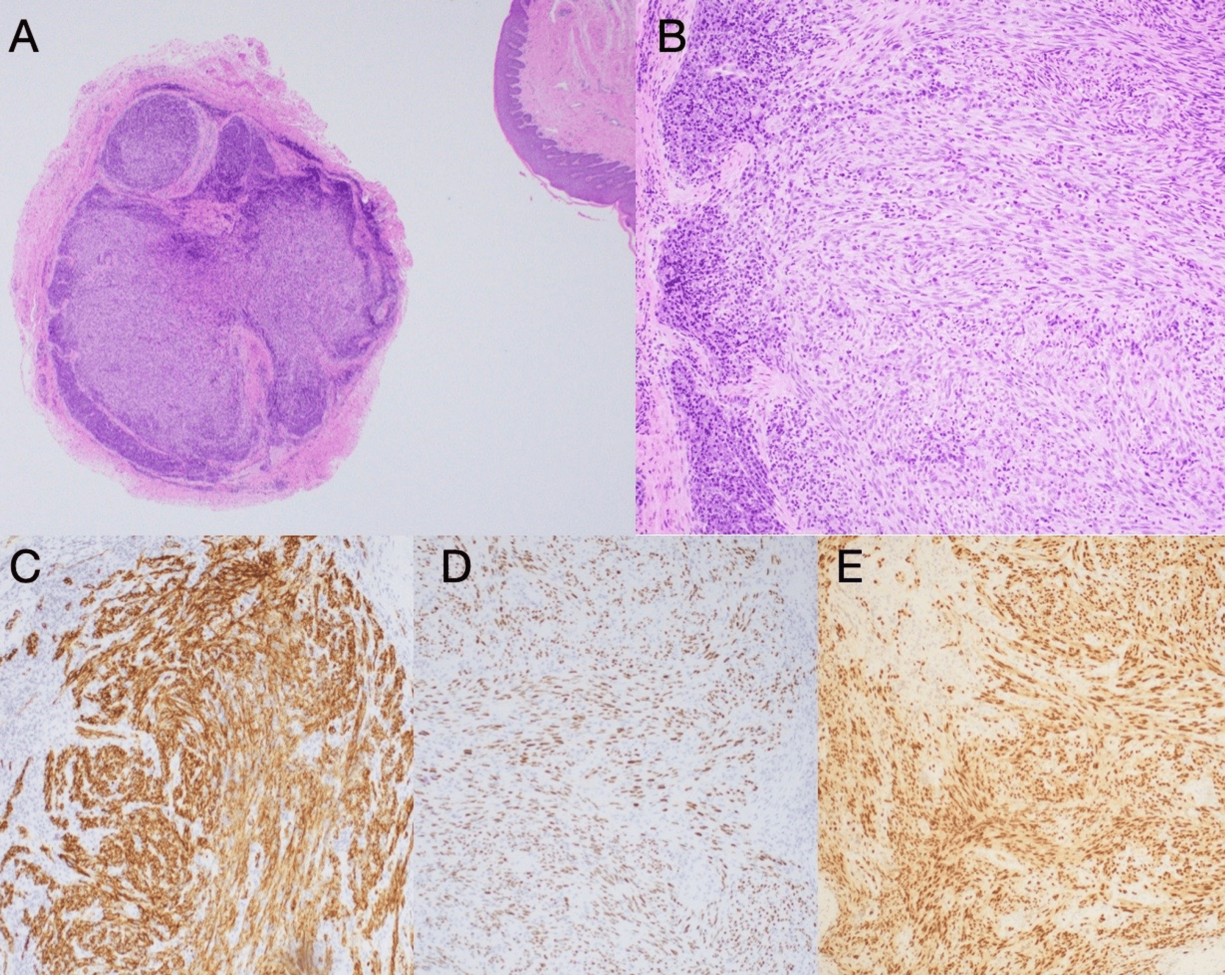
Punch biopsy from penile lesion demonstrating nodular spindle cell proliferation A. H&E at 2x magnification. B. H&E at 10x magnification. C. CD34 stain. D. HHV-8 stain. E. ERG stain H&E: hematoxylin and eosin. CD34: cluster of differentiation 34. HHV-8: human herpesvirus 8. ERG: erythroblast transformation-specific regulated gene

A punch biopsy of the lesion showed a nodular proliferation of dermal spindle cells with associated plasma cells and slit like vascular spaces (Figures 2A, 2B). Margins were negative. Immunohistochemical staining was performed, showing the spindle cells to be positive for CD34, ERG (erythroblast transformation-specific related gene), and HHV-8 LANA1 (latency-associated nuclear antigen one), which confirmed the diagnosis of KS (Figures 2C-2E).

The patient was referred to oncology for further evaluation, and no further workup or intervention was recommended, with a recommendation made for periodic surveillance monitoring. He was also instructed to continue using valacyclovir 500 mg daily and follow up with dermatology every six months for full-body skin examinations. He was educated on the signs and symptoms of KS recurrence with return precautions. This patient was not found to have any evidence of disease at his three-month follow-up appointment.

## Discussion

While KS has been historically associated with four subtypes (HIV-associated, classic, African-endemic, and immunosuppression-associated), it is important to consider the diagnosis in HIV-negative MSM as well, given this more recently recognized fifth epidemiologic risk subgroup. One potential explanation for the timing of the development of KS in this patient is related to his suppressive valacyclovir treatment regimen for chronic orolabial herpes infection. While valacyclovir targets herpes simplex virus, it may have a broader suppressive effect on the human Herpesviridae family given the viruses’ structural similarities. In one study of HHV-8 and HIV coinfected men, controlling for the use of antiretrovirals, daily valacyclovir use was associated with an 18% decrease in oropharyngeal shedding of HHV-8 [5]. We hypothesize that our patient’s discontinuation of valacyclovir shortly before the onset may have allowed for the opportunity for HHV-8 reactivation and subsequent development of KS, although more research into this potential effect is warranted.

Unlike the typical presentation of KS as violaceous nodule(s) or plaque(s), our patient’s lesion was solitary and skin colored, serving as a reminder that KS should not be ruled out by appearance alone in a patient with relevant risk factors. A few prior reports have identified KS presenting in MSM with a solitary penile lesion; however, virtually all of these lesions range in color from red/brown to blue/purple, rather than appearing to be skin-colored [3]. Differential diagnosis for this presentation also includes epidermoid cyst as previously noted, as well as intradermal nevus, fibrous papule, hypertrophic scar, Merkel cell carcinoma, squamous cell carcinoma, basal cell carcinoma, amelanotic melanoma, among other entities. Differential diagnosis for more classic vascular-appearing KS lesions includes, additionally, lymphoma, hemangioma, pyogenic granuloma, bacillary angiomatosis, acroangiodermatitis, and deep fungal infections, among others.

While KS in HIV-positive and immunocompromised patients may follow an aggressive disease course with predilection for lymphatic and visceral involvement, KS in HIV-negative MSM typically presents as a solitary lesion with an indolent course [3,6], though this clinical subtype has rarely been associated with lymphoproliferative disorders [1,2]. In cases of solitary penile KS in an HIV-negative patient, surgical excision of the lesion with negative margins generally is curative, although follow-up surveillance is recommended due to rare reports of recurrence [3].

## Conclusions

KS is a rare, low-grade vascular tumor that is most commonly associated with HIV-positive and immunosuppressed individuals but can occur in any patient infected with HHV-8. We described the case of an HIV-negative male patient identifying as MSM presenting with an atypical and nonspecific-appearing solitary penile lesion of KS confirmed by biopsy. This report highlights the need to maintain clinical suspicion for KS, even in immunocompetent individuals with less typically appearing lesions.
